# Group B *Streptococcus* Infections Caused by Improper Sourcing and Handling of Fish for Raw Consumption, Singapore, 2015–2016

**DOI:** 10.3201/eid2312.170596

**Published:** 2017-12

**Authors:** Man L. Chau, Swaine L. Chen, Min Yap, Sri H.P. Hartantyo, Paul K.T. Chiew, Charlene J. Fernandez, Wai K. Wong, Rockey K. Fong, Wei L. Tan, Brian Z.Y. Tan, Youming Ng, Kyaw T. Aung, Kurosh S. Mehershahi, Christopher Goh, Joanne S.L. Kang, Timothy Barkham, Adeline O.K. Leong, Ramona A. Gutiérrez, Lee C. Ng

**Affiliations:** National Environment Agency, Singapore (M.L. Chau, M. Yap, S.H.P. Hartantyo, Y. Ng, K.T. Aung, C. Goh, J.S.L. Kang, A.O.K. Leong, R.A. Gutiérrez, L.C. Ng);; Genome Institute of Singapore, Singapore (S.L. Chen);; National University of Singapore, Singapore (S.L. Chen, K.S. Mehershahi);; Agri-Food and Veterinary Authority of Singapore, Singapore (P.K.T. Chiew, C.J. Fernandez, W.K. Wong, R.K. Fong, W.L. Tan, B.Z.Y. Tan);; Nanyang Technological University, Singapore (P.K.T. Chiew, L.C. Ng);; Tan Tock Seng Hospital, Singapore (T. Barkham)

**Keywords:** streptococci, group B Streptococcus, GBS, bacteria, outbreak, humans infections, clonal complex, sequence type, handling, consumption, raw fish, food safety, Singapore

## Abstract

We assessed microbial safety and quality of raw fish sold in Singapore during 2015–2016 to complement epidemiologic findings for an outbreak of infection with group B *Streptococcus* serotype III sequence type (ST) 283 associated with raw fish consumption. Fish-associated group B *Streptococcus* ST283 strains included strains nearly identical (0–2 single-nucleotide polymorphisms) with the human outbreak strain, as well as strains in another distinct ST283 clade (57–71 single-nucleotide polymorphisms). Our investigations highlight the risk for contamination of freshwater fish (which are handled and distributed separately from saltwater fish sold as sashimi) and the need for improved hygienic handling of all fish for raw consumption. These results have led to updated policy and guidelines regarding the sale of ready-to-eat raw fish dishes in Singapore.

## Abstract 2

Policies and guidelines regarding sale of ready-to-eat raw fish dishes have been updated.

A major outbreak of group B *Streptococcus* (GBS) infection associated with consumption of a Chinese-style raw fish dish (*yusheng*) occurred in Singapore during 2015 and involved 238 persons during the first half of the year (*1*). The *Yusheng* was typically made from sliced Asian bighead carp (*Hypophthalmichthys nobilis*) and snakehead (*Channa* spp.) and served as a side dish with porridge by food stalls within larger eating establishments. Persons with severe clinical cases had meningoencephalitis, bacteremia, and septic arthritis (*2*–*4*). GBS, or *Streptococcus agalactiae*, was identified as the causative agent (*2*,*3*).

GBS is found in ≈30% of healthy adults (*5*) and is a member of the human commensal gastrointestinal and genitourinary flora (*4*). GBS is a common cause of neonatal sepsis, is acquired by newborns from the vaginal flora of the mother, and is an increasingly common pathogen among vulnerable populations (*6*). The incidence of invasive disease in adults, particularly older adults, has been increasing (*7*,*8*). GBS is also a fish and bovine pathogen (*9*). Although GBS has been shown to colonize the gastrointestinal tract of humans linked to fish consumption (*9*), foodborne transmission leading to invasive disease has not been reported. Local epidemiologic investigations conducted separately (*2*,*3*) identified a single strain of GBS serotype III sequence type (ST) 283 as the causative agent of the outbreak in Singapore during 2015. GBS ST283 had previously been isolated from tilapia in Thailand (*10*) and in adult human cases in Hong Kong (*11*). However, GBS ST283 has not been reported to colonize the human gastrointestinal tract, although to date only 1 study of fish mongers and fish handlers has specifically looked for colonization by this strain (*12*).

We investigated microbial safety and quality of fish sold in the Singapore market during and after the outbreak during 2015 to trace the source of GBS ST283 and provide risk assessment data to support outbreak control and prevention measures. Shortly after identification of GBS ST283 as the cause of the outbreak, these data supported implementing a ban on the sale of ready-to-eat (RTE) dishes containing raw freshwater fish, as well as imposing additional requirements for sale of RTE raw fish dishes made with saltwater fish (*13*). We report the results of our analysis, which might assist the review of guidelines for handling of fish meant for raw consumption in Singapore and other countries. This report offers unique food and environmental insights into the investigation of this outbreak and complements published epidemiologic findings (*2*,*3*).

## Materials and Methods

### Collection of Fish and Fish Tank Water Samples

We collected samples of fish commonly used for raw consumption (n = 997) and fish tank water for holding live freshwater fish (n = 102) along the supply chain in Singapore during August 2015–January 2016 (Technical Appendix Table 1). We tested samples for GBS, *Aeromonas* spp., *Listeria monocytogenes*, *Salmonella* spp., *Vibrio cholerae*, and *V. parahaemolyticus*, and determined *Escherichia coli* counts, *Staphylococcus aureus* counts, and standard plate counts (SPCs) (Technical Appendix). We characterized selected species to determine their virulence potential (Technical Appendix).

### Statistical Analysis

We evaluated significant differences (p<0.05) between bacterial counts (log_10_ CFU/g) and presence of specific foodborne bacteria by using Kruskal-Wallis, Mann-Whitney, χ^2^, and Fisher exact tests as appropriate. We performed analysis by using SPSS version 24.0 software (IBM, Armonk, NY, USA).

## Results

### Raw Fish Samples from Food Stalls and Restaurants/Snack Bars

Although raw freshwater and saltwater fish were served as RTE food at food stalls, only raw saltwater fish were reportedly served at restaurants/snack bars. PCR positivity rates were 43.5% (20/46) for GBS and 23.9% (11/46) for GBS serotype III in sliced fish samples from food stalls. Fish sampled from restaurants/snack bars had significantly lower rates (p<0.05) of 9.2% (26/282) for GBS and 0.7% (2/282) for GBS serotype III (Table). Among the 20 GBS PCR-positive samples from food stalls, 5 yielded isolates; these isolates were of serotype II ST652, serotype III ST283, serotype III ST335, and serotype V ST1 (Technical Appendix Table 3). The GBS ST283 isolated was from a RTE sliced fish sample sold as grass carp collected from a food stall linked to a human case, as described (*12*). We did not detect GBS ST283 in samples from restaurant/snack bars; however, we did find a range of other GBS, including serotypes Ia ST7, Ia ST103, Ia ST485, III ST651, III ST861, V ST1, V ST24, VI ST167, and VII ST1.

We found *Salmonella* serogroup B ST29 (serovar Stanley) (n = 2); *V. parahaemolyticus* (negative for *tdh*, *trh1*, and *trh2* genes) (n = 1); and non–O1 *V. cholerae* (n = 1) in freshwater fish samples from food stalls. We also isolated *V. cholerae* from saltwater fish samples, 1 from a food stall and 1 from a restaurant. We detected *L. monocytogenes* in 5 samples from restaurants/snack bars.

**Figure 1:**
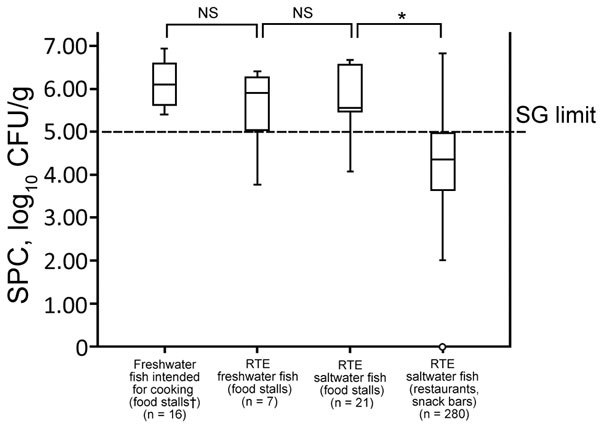
SPCs for sliced fish samples collected from various eating establishments during investigation of group B *Streptococcus* infections, Singapore, 2015–2016. Dashed horizontal line indicates regulatory limit of Singapore for SPCs for ready-to-eat foods (<5 log_10_ CFU/g) (*14*). Top and bottom of boxes in plots indicate 25th and 75th percentiles, horizontal lines indicate medians, and whiskers indicate minimum and maximum values. *p<0.05. †Food stalls housed within larger eating establishments that include hawker centers, coffee shops, and eating houses. Open circle indicates an outlier. NS, not significant (p>0.05); RTE, ready to eat; SG, Singapore government; SPCs, standard plate counts.

SPCs of most RTE raw freshwater (71.4%, 5/7) and saltwater (85.7%, 18/21) fish samples from food stalls exceeded the regulatory limit for RTE food (5 log_10_ CFU/g) in Singapore (*14*). We observed no difference in SPCs for fish slices intended for raw consumption and cooking purposes (Figure 1). We also found that 24.8% (70/282) of saltwater fish samples from restaurants/snack bars did not comply with regulatory limits for SPCs, *E. coli* counts (1.3 log_10_ CFU/g), or both (*14*). These results showed the poor quality of RTE raw freshwater and saltwater fish sold at food stalls in comparison to those sold at restaurants and snack bars.

### Comparison of Freshwater and Saltwater Fish Samples from Fresh Produce Markets

Fish sold at food stalls were typically procured from local fresh produce markets. For the 62 samples of whole fish and fish parts we collected from these markets, we detected GBS ST283 in 28.2% (11/39) of the freshwater fish (Table), which included fish sold as tilapia, Asian bighead carp, grass carp, snakehead-haruan, snakehead-toman, and silver carp (Technical Appendix Table 3). However, we did not detect GBS ST283 in saltwater fish. Other GBS strains detected among these fish include serotypes Ia ST7, Ia ST23, Ia ST24, and II ST28 (Technical Appendix Table 3).

**Table:** Positivity rates for GBS and other foodborne bacteria in fish samples, Singapore, 2015–2016*

Characteristic	Targeted bacteria, no. positive samples/no. tested (%)									
	All GBS	GBS serotype III	GBS serotype III ST283	*Aeromonas* spp.†	*E. coli*	*S. aureus*	*V. c.*	*V. p.*	*L. m.*	*Salmonella* spp.
Detection method	PCR	PCR	Culture, PCR	Culture	Culture	Culture	Culture	Culture	Culture	Culture
Ports										
Freshwater fish, n = 586	27/586 (4.6)	12/586 (2.0)	6/586 (1.0)	NT	NT	NT	NT	NT	NT	NT
Fresh produce markets‡										
Freshwater fish, n = 39	30/39 (76.9)^a^	14/39 (35.9)^b^	11/39 (28.2)	16/39 (41.0)	32/39 (82.0)^c^	11/39 (28.2)	6/39 (15.4)	2/39 (5.1)	0/39	0/39
Saltwater fish, n = 23	5/23 (21.7)^a^	2/23 (8.7)^b^	0/23	14/23 (60.9)	8/23 (34.8)^c^	6/23 (26.1)	2/23 (8.7)	2/23 (8.7)	0/23	0/23
Sashimi suppliers§										
Saltwater fish, n = 21	0/21	0/21	0/21	10/21 (47.6)	1/21 (4.7)^c^	0/21	0/21	0/21	1/21 (4.7)	0/21
Food stalls¶										
RTE freshwater fish, n = 7	5/7 (71.4)	4/7 (57.1)	1/7 (14.3)	NT	0/7	0/7 (0)	0/7	0/7	0/7	1/7 (14.3)
Freshwater fish for cooking, n = 18	8/18 (44.4)	4/18 (22.2)	0/18	NT	2/18 (11.1)	0/18	1/18 (5.6)	1/18 (5.6)	0/18	1/18 (5.6)
RTE saltwater fish, n = 21	7/21 (33.3)^d^	3/21 (14.3)^e^	0/21	NT	0/21	0/21	1/21 (5.0)	0/21	0/21	0/21
Restaurants, snack bars										
RTE saltwater fish, n = 282	26/282 (9.2)^d^	2/282 (0.7)^e^	0/282	NT	0/282	0/282	1/282 (0.4)	0/282	5/282 (1.8)	0/282

*A sample was considered positive when a specific organism was detected in >1 subsamples (surface, muscle, or organs) of a fish sample. Superscript letters a–e indicate a significant difference (p<0.05) in positivity rates of targeted bacteria between fish types. *E. coli, Escherichia coli*; GBS, group B *Streptococcus*; *L. m.*, *Listeria monocytogenes*; NT, not tested; RTE, ready to eat; *S. aureus*, *Staphylococcus aureus*; ST283, sequence type 283; *V. c.*, *Vibrio cholerae*; *V. p.*, *V.* *parahaemolyticus*. †*Aeromonas caviae*, *A. hydrophila*, and *A. sobria*. ‡Fish stalls at ports and wet markets, and fresh produce sections of supermarkets, excluding sashimi and sushi counters of supermarkets. §Companies that supplied sashimi-grade fish to restaurants and snack bars. ¶Within larger eating establishments that include hawker centers, coffee shops, and eating houses.

We detected *Aeromonas* spp. (48.4%, 30/62), *S. aureus* (27.4%, 17/62), non–O1 *V. cholerae* (12.9%, 8/62) and *V. parahaemolyticus* (negative for *tdh*, *trh1*, and *trh2* genes) (6.4%, 4/62) in fish samples from fresh produce markets. There was no difference in positivity rates of these organisms between freshwater and saltwater fish. We did not detect *L. monocytogenes* or *Salmonella* spp. in any fish samples collected from fresh produce markets.

**Figure 2:**
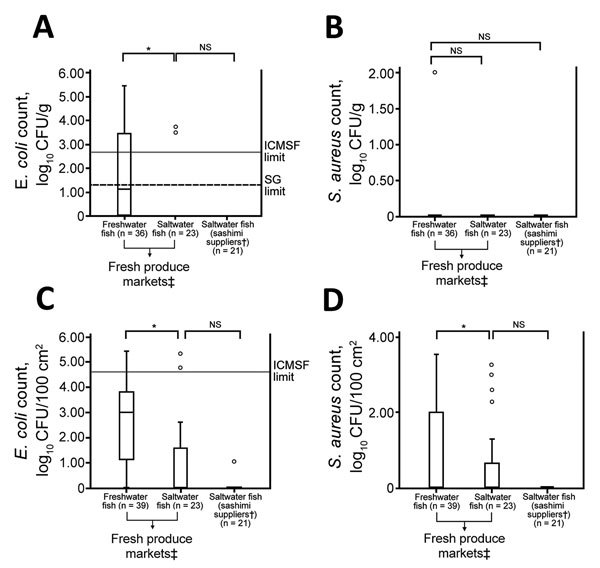
*Escherichia coli* (A and C) and *Staphylococcus aureus* (B and D) counts in fish samples (muscle and surface swabs) collected from fresh produce markets during investigation of group B *Streptococcus* infections, Singapore, 2015–2016. Solid horizontal lines indicate ICMSF limit for *E. coli* count in fresh fish intended for cooking (<2.7 log_10_ CFU/g or <4.7 log_10_ CFU/100 cm^2^) (23). Dashed horizontal line indicates Singapore regulatory limit for *E. coli* count ready-to-eat foods (<1.3 log_10_ CFU/g) (*14*). Top and bottom of boxes in plots indicate 25th and 75th percentiles, horizontal lines indicate medians, and whiskers indicate minimum and maximum values. Open circles indicate outliers. *p<0.05. †Companies that supplied sashimi grade fish to restaurants and snack bars. ‡Fish stalls at ports and wet markets, as well as fresh produce sections of supermarkets, excluding sashimi and sushi counters of supermarkets. ICMSF, International Commission on Microbiological Specifications of Foods; NS, not significant (p>0.05); SG, Singapore government; SPCs, standard plate counts.

Approximately 42% (15/36) of freshwater fish muscle samples had SPCs or *E. coli* counts, or both, exceeding regulatory limits for RTE food in Singapore (*14*). Positivity rates for GBS, GBS serotype III, and *E. coli*, as well as SPCs for saltwater fish, were significantly lower (p<0.05) (Figure 1; Table). *E. coli* and *S. aureus* counts for freshwater fish surfaces were significantly higher (p<0.05) than those for saltwater fish (Figure 2).

We collected 4 fish tank water samples from wet markets and supermarkets. One water sample and the live freshwater fish the tank contained were positive for GBS by PCR and non–O1 *V. cholerae* by culture; the associated fish was positive for GBS ST283 by culture. Two other fish tank water samples and the live fish the tanks contained were positive for *E. coli*, *S. aureus*, or both. The level of *E. coli* detected in each positive fish tank water sample was 1.3 log_10_ CFU/500 mL, which was greater than the 1 log_10_ CFU/500 mL coliform (which includes *E. coli*) limit set by the British Columbia Centre for Disease Control (*15*).

### Whole Freshwater Fish and Fish Tank Water from Ports

We tested for GBS only in whole fish and fish tank water samples collected from ports. We detected GBS ST283 in 1% (6/586) of freshwater fish samples; positive samples were from Asian bighead carps imported from and farmed in Malaysia. For 98 fish tank water samples collected from ports, 55.1% (54/98) were positive for GBS, and 6.1% (6/98) were positive for GBS ST283. Three of the GBS ST283–positive fish were kept in fish tank water that was also positive for GBS ST283.

### Comparison of Saltwater Fish from Fresh Produce Markets and Sashimi Suppliers

**Figure 3:**
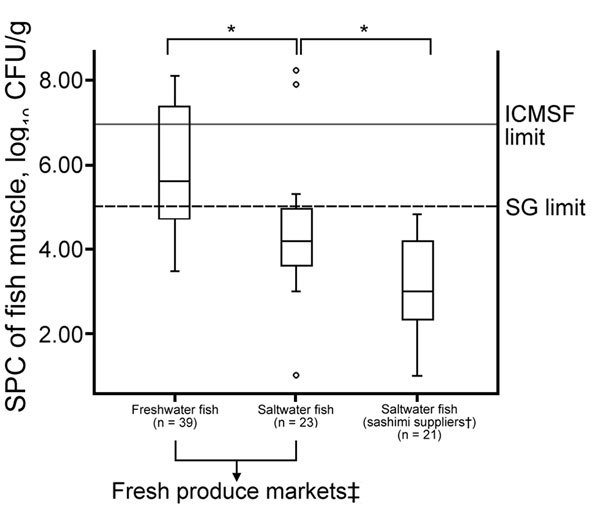
SPCs for fish samples (muscle) collected from fresh produce markets during investigation of group B *Streptococcus* infections, Singapore, 2015–2016. Solid horizontal line indicates ICMSF limit for SPCs in fresh fish intended for cooking (<7 log_10_ CFU/g) (*23*). Dashed horizontal line indicates Singapore regulatory limit for SPCs for ready-to-eat foods (<5 log_10_ CFU/g) (*14*). Top and bottom of boxes in plots indicate 25th and 75th percentiles, horizontal lines indicate medians, and whiskers indicate minimum and maximum values. Open circles indicate outliers. *p<0.05. †Companies that supplied sashimi grade fish to restaurants and snack bars. ‡Fish stalls at ports and wet markets, as well as fresh produce sections of supermarkets, excluding sashimi and sushi counters of supermarkets. ICMSF, International Commission on Microbiological Specifications of Foods; SG, Singapore government; SPCs, standard plate counts.

Our data indicate the risk for contamination of fish sold at local fresh produce markets, although saltwater fish samples from fresh produce markets had lower rates of contamination than freshwater fish samples. The SPCs and the positivity rates for *E. coli* in saltwater fish samples from sashimi suppliers were significantly lower (p<0.05) than those for saltwater fish samples from fresh produce markets (Figure 3; Table), which suggested that the microbial quality of fish could be managed by improvements in handling throughout distribution channels. None of the saltwater fish muscle samples from sashimi suppliers exceeded the Singapore SPC (5 log_10_ CFU/g) and *E. coli* (1.3 log_10_ CFU/g) limits for RTE food (*14*). We did not detect GBS, *S. aureus*, *V. cholerae*, and *V. parahaemolyticus* in any fish samples collected from sashimi suppliers. However, we detected *L. monocytogenes* in 1 salmon sample.

### Characterization of GBS Isolates

We detected 6 GBS serotypes (Ia, II, III, V, VI, and VII) and 13 STs (1, 7, 23, 24, 28, 103, 167, 283, 335, 485, 651, 652, and 861) in fish (Technical Appendix Table 3). Although most strains were within clonal complexes (1, 10, 17, 19, and 23) associated with human carriage and diseases (*16*), a total of 20 isolates from 7 sashimi samples (SGEHI2015-IV45, SGEHI2015-IV72, SGEHI2015-IV74, SGEHI2015-IV89, SGEHI2015-IV100, SGEHI2015-IV211, and SGEHI2015-IV232) did not belong to these clonal complexes. These strains had few closely related strains in the public genomic databases, and the closely related strains are mostly from animals (Technical Appendix Figure 2).

**Figure 4:**
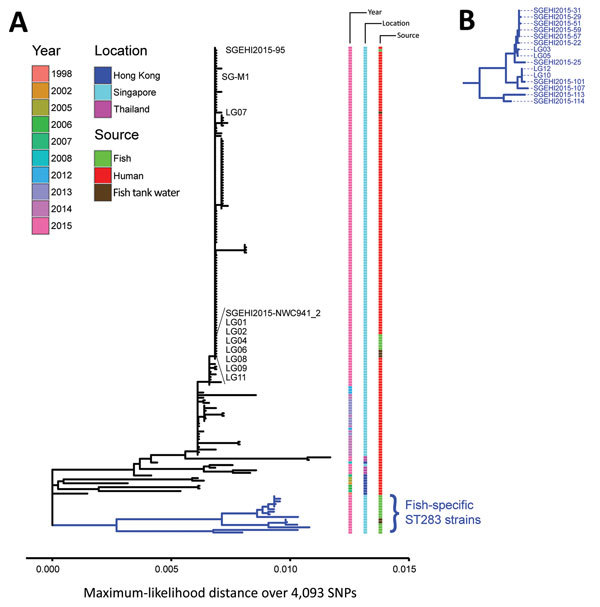
Phylogenetic analysis of group B *Streptococcus* (GBS) infections caused by improper sourcing and handling of raw fish for raw consumption, Singapore, 2015–2016. A) Maximum-likelihood single-nucleotide polymorphism (SNP)–based tree for GBS ST283 strains relative to the SG-M1 reference human outbreak strain. Year, location, and source (human or fish) for isolates are indicated. Twelve strains from 6 fish (SGEHI2015-NWC941, SGEHI2015–95, LG01, LG02, LG04, and LG06) and 4 fish tank water samples (LG07, LG08, LG09, and LG11) were nearly identical to the local reference outbreak strain SG-M1 (no SNP, 0 and 12 indels, respectively). Scale bar indicates distance over 4,093 total SNPs. B) Enlargement of blue subtree from bottom of tree in panel A showing fish GBS ST283 isolates that were different (57–71 SNPs and 11–33 indels) from the human outbreak strain. ST, sequence type.

We found GBS ST283 only among freshwater fish and water for holding freshwater fish. Genomic analyses indicated that GBS ST283 isolated from fish clustered in 2 clades (Figure 4). The first clade included 12 isolates from 6 fish from a food stall, a fresh produce market and a port, and 4 fish tank water samples from a port. Genome sequencing showed that these 12 isolates were nearly identical (0–2 SNPs and 0, 1, and 12 indels all in homopolymeric runs of >4 nt) compared with the 2.1-Mbp genome of the reference human outbreak strain, SG-M1, isolated from a meningitis patient during the GBS outbreak in Singapore during 2015 (*12*,*17*). Isolates that clustered into the second clade were 20 isolates from 12 fish and 2 fish tank water samples and did not include any human isolates either from this outbreak or from previous reports of human GBS infecting isolates. Sequences of these isolates showed higher intraclade diversity (57–71 SNPs and 11–33 indels) when compared with the SG-M1 genome (Figure 4).

### Characterization of *S. aureus, V. cholerae*, and *V. parahaemolyticus* Isolates

We characterized 18 *S. aureus* isolates from 17 fish. All except 1 were obtained from fish surfaces. We detected >1 enterotoxin gene in two thirds of these isolates and the *sec* gene in 55.6% (10/18) of the isolates. Other enterotoxin genes (*sea*, *seg*, *seh*, *sei*, and *sel*) were detected at much lower rates (5.6% [1/18] to 11.1% [2/18]). We detected 4 enterotoxin genes (*sec*, *seg*, *sei*, and *sel*) in a *S. aureus* isolate obtained from the surface of a wolf herring sample collected from a port. We did not detect virulence genes (*ctxA*, *ctxB*, and *tcpA*) in any of the 16 non–O1 *V. cholerae* isolates from 9 fish and 1 fish tank water samples and did not detect virulence genes (*tdh*, *trh1*, and *trh2*) in any of the 6 *V. parahaemolyticus* isolates from 5 fish samples.

## Discussion

We found GBS ST283, the causative strain of a severe foodborne outbreak in Singapore, in the local freshwater fish supply chain that stretches from food stalls to local fresh produce markets and back to ports. Patients with GBS ST283 infections during this outbreak were more likely to show development of meningoencephalitis, septic arthritis, and spinal infection than were persons with non–GBS ST283 infections (*12*). Although this study suggested Malaysia as a source of the strain, the finding of the same ST in Hong Kong and Thailand (*10*,*11*) suggested that GBS ST283 is generally prevalent throughout the region.

Our analysis shows that there are at least 2 clades of GBS ST283 strains among fish in local markets. Fish and water strains from 1 clade were nearly identical to clinical strains from this outbreak (Figure 4). The small variability of 0–2 SNPs and 0–12 indels between fish and water strains and the reference human outbreak strain (SG-M1) is equivalent to variability observed in 131 clinical strains from the same outbreak reported elsewhere (0–5 SNPs from the SG-M1 reference) (*12*). Strains from a second clade of GBS ST283 had a difference of 57–71 SNPs and 11–33 indels when compared with the SG-M1 genome. Other GBS ST283 isolates, many collected in Hong Kong <17 years before this outbreak (*11*) are also different from the SG-M1 strain (<129 SNPs) (Figure 4). We found no human-infecting isolate from Singapore or elsewhere within the second fish-associated GBS ST283 clade.

A major issue is whether all GBS ST283 strains are capable of causing invasive human disease by the foodborne route. If strains from the fish-associated clade are not pathogenic to humans, they could be used as effective controls for identifying the genetic basis of pathogenicity of the first clade and the cause of its emergence, which resulted in outbreak in Singapore in 2015. If these strains are pathogenic to humans, then broader tracking of the prevalence of GBS ST283 would be warranted.

In contrast to GBS strains that are known to cause disease outbreaks in fish (*10*,*18*), the live and whole fish from which GBS ST283 was recovered in this study did not have defects, such as corneal opacity and exophthalmia (*18*), which suggests that this ST might not be pathogenic for freshwater fish. The closest GBS fish pathogens with published genomes, GD201008–001 (*19*) and HN016 (*20*), are serotype Ia ST7 strains that are distant (>4,000 SNPs) from all ST283 strains that our group and others have identified (*12*).

Detection of 6 GBS serotypes and 13 STs showed the diversity of GBS strains in fish. Although the sample size in this study was small and our results might not represent the distribution of GBS in all fish species, our findings provide valuable data for characterizing the public health risk from consuming raw fish. No baseline information on GBS in fish was publicly available before this outbreak because fish were not a recognized source or a recognized route of transmission of GBS. Further work on GBS STs other than ST283 is underway to investigate the role of fish as a source of GBS disease in humans.

Several GBS strains from sashimi had relatively few closely related strains in the public genomic databases (Technical Appendix Figure 2), which suggests that the GBS population associated with saltwater fish could be different from that associated with freshwater fish and humans. Another reason for this observation is that GBS from food and environmental sources are relatively undersampled in the genomic databases than those from humans.

We detected GBS serotypes Ia ST23 and Ia ST7, which are associated with human carriage (*10*), in fish samples. Although GBS ST7 has been described as a fish pathogen, the presence of GBS serotype Ia ST23 has not been reported in fish (*10*). GBS serotypes Ia ST23, and Ia ST7 and *E. coli*, which are all associated with human gut flora, suggest possible contamination of fish by effluent water.

The intentional introduction of animal feces into fish ponds as part of integrated farming (*21*,*22*) might further contribute to the complex flow of pathogens between animals and humans. Such findings point to areas for research to clarify the diversity and role of GBS strains in affecting animal and human health. For instance, GBS ST861, which was isolated from salmon in this study (Technical Appendix Table 3), was also isolated from a clinical case in the same year in Singapore on the basis of metadata available in the PubMLST *S. agalactiae* database (http://pubmlst.org/sagalactiae/).

In addition to the finding of GBS ST283 in freshwater fish, detection of high SPCs and *E. coli* and *S. aureus* counts indicates the hazard of using such fish for preparing raw RTE dishes. Because *E. coli* is not part of the intestinal flora of cold-blooded animals (*23*), its presence suggests contamination from polluted water, unhygienic handling, or temperature abuse after harvesting. Similarly, because *S. aureus* is not part of usual fish flora, its presence on fish surfaces suggests possible transfer of human skin flora caused by unhygienic handling (*24*). We detected *V. parahaemolyticus*, an organism known to grow well in seawater but lyse rapidly in freshwater (*24*), in freshwater fish samples from fresh produce markets (5.1%, 2/39). This finding was not surprising because freshwater and saltwater fish are typically sold, handled, stored, and degutted within the same confined areas in fresh produce markets. Thus, despite lower SPCs and positive rates for *E. coli* in saltwater fish than in freshwater fish from fresh produce markets, saltwater fish procured from such environments are prone to cross-contamination.

Fish used by food stalls were generally obtained from such markets. Moreover, microbial counts for sliced fish samples from eating establishments indicated that most food stalls were not able to prepare RTE raw fish dishes of acceptable hygiene quality. Poor practices observed included use of common chopping boards, knives, or slicers for preparing fish slices meant for raw consumption and cooking. If fish slices are contaminated, rinsing with water cannot improve their quality (Technical Appendix).

In contrast to the quality of saltwater fish samples from fresh produce markets, all saltwater fish samples from sashimi suppliers complied with local SPCs (5 log_10_ CFU/g) and *E. coli* (1.3 log_10_ CFU/g) limits for ready-to-eat food (*14*); all samples were negative for GBS, *S. aureus*, *Salmonella* spp., and *V. parahaemolyticus*. The compliance rate among restaurants/snack bars was higher because such premises are more likely to procure fish from sashimi suppliers that harvest fish from cleaner waters and adhere to stricter cold chain management practices. However, some saltwater fish samples from sashimi suppliers and restaurants were found to contain *Aeromonas* spp. (47.6%, 10/21) and *L. monocytogenes* (2.0%, 6/303), whose psychrotrophic nature has posed a challenge to the fish industry. *L. monocytogenes* is also a concern in chilled RTE food because of its ubiquity and persistence in food-processing environments (*25*).

Food and environmental findings of our study were consistent with epidemiologic findings for this outbreak (*2*,*3*). Multivariate analyses of a case–control study showed that persons who had consumed *yusheng* were more likely to acquire GBS ST283 infections than those who had not consumed *yusheng* (*2*). However, there was no strong association between GBS ST283 infections and consumption of sashimi and sushi (*2*).

Findings of this study have led to implementation of new policies in Singapore. These new policies included banning the use of freshwater fish in RTE dishes and requiring procurement of saltwater fish from suppliers for raw fish approved by the Agri-Food and Veterinary Authority of Singapore. Food stalls and food establishments providing catering services were required to stop selling RTE raw fish dishes until they complied with practices required for preparing RTE raw saltwater fish dishes.

The number of RTE fish samples collected from food stalls was limited because eating establishments were advised to stop the sale of RTE raw fish dishes containing Asian bighead carp and snakehead during July 24–December 5, 2015, while the outbreak investigation was underway (*1*). Sampling was not random because it was part of an outbreak investigation, but it was biased toward fish species and food stalls implicated in the outbreak. Thus, contamination rates might not reflect contamination rates of all fish species sold for raw consumption in the market. Similarly, testing of samples from ports and retail outlets was performed by using different protocols, which limited comparisons that could be made.

In conclusion, we detected GBS ST283, which caused a severe foodborne outbreak in Singapore in 2015, in freshwater fish, not only in food stalls and fish markets, but also in ports from which fish are imported. Comparison of human and fish isolates showed as few as 0–2 SNPs between human and fish isolates of GBS ST283 on a background of a diversity of GBS and other bacteria in freshwater fish. These data indicate the risk for contamination of raw freshwater fish and underscore the need for proper sourcing and handling of all fish for raw consumption. To control the outbreak, a ban on the sale of RTE raw freshwater fish dishes was implemented, and additional requirements were imposed for the sale of RTE raw fish dishes made with saltwater fish (*13*). Our study complements the epidemiologic findings for this outbreak (*2*,*3*) and illustrates the need for public health authorities and industries to remain vigilant regarding emerging pathogens.

Technical Appendix Additional information on Group B *Streptococcus* infections caused by handling and consumption of raw fish, Singapore, 2015–2016.
