# Supplementary material for: Group B Streptococcus Infections Caused by Improper Sourcing and Handling of Fish for Raw Consumption, Singapore, 2015–2016
Source: Emerg Infect Dis. 2017 Dec;23(12):2002–10. doi: 10.3201/eid2312.170596 (PMC5708258; doi:10.3201/eid2312.170596)
Supplement: Technical Appendix — Additional information on Group B Streptococcus infections caused by handling and consumption of raw fish, Singapore, 2015–2016. [file 17-0596-Techapp-s1.pdf]

Article DOI: https://doi.org/10.3201/eid2312.170596

Group B *Streptococcus* Infections Caused by Improper Sourcing and Handling of Fish for Raw Consumption, Singapore, 2015–2016

Technical Appendix

Materials and Methods

Collection of Fish and Fish Tank Water Samples

Samples from Eating Establishments

Sliced fish samples (n = 328) from food stalls, restaurants, and snack bars were analyzed for microbiological safety and quality between freshwater and saltwater fish slices prepared by these retail eating establishments. Eating establishments linked to human cases (implicated establishments) and eating establishments that sold similar dishes but were not linked to human cases were included (control establishments). However, statistical analyses for implicated and control establishments were not performed because the number of samples from implicated establishments was small (26 food stall samples; 5 restaurant/snack bar samples). Limited samples were available because retailers were advised by health authorities to stop selling ready-to-eat raw fish dishes containing Asian bighead carp and snakehead while the outbreak investigation was underway.

Samples from Fresh Produce Markets and Sashimi Suppliers

Although fresh produce markets refer to fish stalls at the ports and wet markets, as well as fresh produce sections of supermarkets that food stalls are known to procure fish from, sashimi suppliers refer to companies that local restaurants and snack bars are known to procure sashimi grade fish from. Fish parts or whole fish (n = 83) were analyzed for microbiological safety and quality for freshwater and saltwater fish sampled at these locations. Subsamples consisting of surface swab specimens, muscles, or organs, were obtained from each whole fish or

fish part sample and subjected to microbial analyses (online Technical Appendix Table 2). During sampling of fish at fresh produce markets, samples of water in which live freshwater fish were kept were also collected. Microbial safety and quality of water were analyzed to assess their potential to cause cross-contamination among live fish in the market.

Samples from Ports

A total of 586 whole freshwater fish samples and 98 holding water samples were collected at ports in Singapore. These samples were tested for group B *Streptococcus* (GBS), GBS serotype III, and GBS sequence type (ST) 283.

Transport of Samples

Whole fish and fish part samples were purchased in their original packaging; sliced fish were collected in sterile bags, and fish tank water samples were collected in sterile bottles. All samples were transported on ice, kept refrigerated in laboratories, and analyzed on the day of collection.

Testing of Samples

Microbiological testing of fish and fish tank water samples was performed by 3 laboratories: the Environmental Health Institute (EHI) of the National Environment Agency, the Veterinary Public Health Laboratory of the Agri-Food and Veterinary Authority of Singapore (AVA), and an accredited commercial laboratory. The Veterinary Public Health Laboratory performed testing for GBS in whole fish and fish tank water samples collected from ports, and EHI performed testing for GBS on all other samples. Standard plates counts (SPCs) for *Escherichia coli*, *Staphylococcus aureus*, *Listeria monocytogenes*, *Salmonella* spp., *Vibrio cholerae*, and *V. parahaemolyticus* from fish slices collected from eating establishments was performed by an accredited commercial laboratory. SPCs for *E. coli*, *S. aureus*, *Aeromonas* spp., *L. monocytogenes*, *Salmonella* spp., *V. cholerae*, and *V. parahemolyticus* in whole fish and fish parts collected from ports, wet markets, supermarkets, and sashimi suppliers was performed by EHI. *S. aureus*, *Salmonella* spp., *V. cholerae*, and *V. parahaemolyticus* isolated by EHI and the accredited commercial laboratory were further characterized at EHI.

GBS Analysis

Fish and fish tank water samples collected from ports were analyzed by Agri-Food and Veterinary Authority of Singapore by using a proprietary method that involved screening for GBS and GBS serotype III with a multiplex PCR method and isolation of viable GBS ST283 by culture method. GBS analyses on all other samples were conducted at EHI by using a modified protocol (*1*). Each fish surface swab sample was prepared by applying a premoistened sponge swab (3M Company, Maplewood. MN, USA) onto the entire surface area of each whole fish or fish part sample. The sponge swab was then suspended in 9 mL of Todd Hewitt broth (Acumedia, Lansing, MI, USA) containing colistin sulfate (7.5 μg/mL) and nalidixic acid (5 μg/mL) and homogenized by using a Stomacher (model 400; Seward Medical, Worthing, UK) with a paddle speed of 230 rpm for 30 s. A total of 25 g each of fish muscle sample, fish organ composite sample, and sliced fish sample was homogenized with 225 mL of Todd Hewitt broth containing antimicrobial drugs by using a Stomacher.

Each 500-mL fish tank water sample was concentrated by using membrane filtration with sterile 0.45-μm pore size filters made of mixed cellulose esters (Merck Millipore, Darnstadt, Germany). Membranes were then suspended in 20 mL of Todd Hewitt broth containing antimicrobial drugs. Homogenized aliquots and membranes were incubated at 37°C for 24 h. Secondary enrichment was performed by transferring 1 mL of the enriched Todd Hewitt broth to 5 mL brain heart infusion broth (Acumedia) and incubating at 37°C for 24 h. Nucleic acids were extracted from enriched brain heart infusion broth and selected preenriched sample suspensions in Todd Hewitt broth containing antimicrobial drugs by using a method described (*2*).

GBS was detected by using a singleplex PCR specific for the *dltR* gene (*3*). All GBS PCR-positive samples were cultured to obtain viable isolates. Culture involved plating of 100 μL of serially diluted ($0–10^5$ dilutions) enriched brain heart infusion broth onto Brilliance GBS Agar (Thermo Fisher Scientific, Darmstadt, Germany) and incubating the agar at 37°C for 24 h. Presumptive mauve colonies were confirmed as GBS by serologic analysis using the Streptococcal Grouping Kit (Oxoid, Basingstoke, UK) according to the manufacturer's instructions.

Although 103 of 522 enriched samples tested by EHI were positive for GBS by PCR, only 35 of these samples yielded GBS isolates when plated on chromogenic agar; GBS was not

recovered from the other 68 PCR-positive samples. A high background flora could have masked GBS in these 68 enriched samples. This masking is supported by the observation that of the 68 isolate-negative samples, 57 initially showed PCR-negative results, but then showed PCR-positive results after a 2-day enrichment. This change in results indicated low amounts of GBS in the original samples. Presampling manipulation, such as freezing, could have also contributed to the low yield.

Because recovery of viable isolates was not always successful for GBS PCR-positive samples, the enriched broth of these samples was further screened for GBS serotype III by using published primers (*4*). This screening was performed to estimate the highest possible contamination rate of GBS serotype III ST283 in fish.

A total of 84 GBS isolates were characterized by using serotyping, multilocus sequence typing (MLST), and whole-genome sequencing methods. Serotyping of GBS isolates was performed by using the IMMULEX STREP-B Antisera Set (Staten Serum Institut Diagnostica, Hillerød, Denmark) according to the manufacturer’s instructions. Results were confirmed by using a published multiplex PCR protocol (*5*). MLST was performed by using published primers and Sanger sequencing (*6*). Aligned nucleotide sequences of amplified products were compared with sequences in the PubMLST *Streptococcus agalactiae* Database (http://pubmlst.org/sagalactiae/) for identification of sequence types.

Whole-genome sequencing was performed by the Genome Institute of Singapore and the Agency for Science, Technology and Research by using a NextSeq 500 sequencer (Illumina, San Diego, CA, USA) according to protocols described (*7*). A total of 1 mL of each GBS overnight culture in brain heart infusion broth was centrifuged, and the pellet of bacterial cells was lysed by using enzymatic lysis buffer at 37°C for 45 min, followed by extraction using the DNeasy Blood and Tissue Kit (QIAGEN, Valencia, CA, USA) according to the manufacturer’s instructions. Genomic DNA shearing was performed by using an M220 Focused Ultrasonicator (Covaris, Woburn, MA, USA), and library preparation was performed by using the TruSeq Nano DNA LT Library Prep Kit Pooled libraries (Illumina). Samples were then sequenced by using a NextSeq 500 sequencer with 2 × 151-bp reads. All sequencing data are available in GenBank under BioProject PRJNA293392.

Sequence Analysis

All primary sequence analysis was performed by the Genome Institute of Singapore with the Efficient Rapid Microbial Sequencing Platform. Reference-based analyses were performed by using the SG-M1 genome (*8*) as a reference. FASTQ files were mapped by using Burrows-Wheeler Aligner version 0.7.10 software (*9*). Indel realignment and single-nucleotide polymorphism (SNP) calling was performed by using Lofreq* version 2.1.2 with default parameters (*10*). Maximum-likelihood SNP trees (ignoring indels) of GBS ST283 strains were created by using SNPhylo (*11*). Neighbor-joining trees of all GBS strains were created by using APE version 3.5 (*12*). All phylogenetic trees were visualized with GGTREE version 3.2 (*13*) in R version 3.2.2 (https://www.R-project.org). Publicly available sequences of GBS were downloaded from the GenBank Short Read Archive (for Illumina datasets; http://www.ncbi.nlm.nih.gov/sra) and the Genbank FTP site (for assembled genomes; https://www.ncbi.nlm.nih.gov/genome/genomes/186?). MLST and resistance gene predictions were made by using SRST2 version 0.1.8 (*14*) for Illumina sequenced strains or manually by using BLASTN (*15*) for fully assembled reference sequences, the recommended MLST database (http://pubmlst.org/sagalactiae/) (*16*), and the ARGannot resistance gene database (*17*) included with SRST2.

SPCs and *E. coli* and *S. aureus* Counts

The protocols used by EHI are described below. Each fish surface swab sample was prepared by applying a premoistened sponge swab onto a 10 cm × 10 cm surface area of each fish part or whole fish sample. The sponge swab was then suspended in 9 mL Butterfield buffer and homogenized by using a Stomacher. A total of 25 g of each fish muscle and sliced fish sample was homogenized with 225 mL of universal preenrichment broth (Acumedia) by using a Stomacher. Each 500-mL fish tank water sample was concentrated by membrane filtration using 0.45-µm pore size filters and suspended in 20 mL of universal preenrichment broth. SPCs and *E. coli* and *S. aureus* counts were determined performed according to described methods (*18*). The presence of the methicillin resistance gene and enterotoxin-producing genes (*sea*, *seb*, *sec*, *sed*, *see*, *seg*, *seh*, *sei*, *sej*, and *sel*) of 18 *S. aureus* isolates from 17 fish was determined by using published primers (*19–21*).

Detection of *Aeromonas* spp., *Listeria monocytogenes, Salmonella* spp., *Vibrio cholerae*, and *V. parahaemolyticus*

Each fish surface swab sample was prepared by applying a premoistened sponge swab onto the entire surface area of each fish part or whole fish sample. The sponge swab was then suspended in 9 mL of universal preenrichment broth and homogenized by using a Stomacher. All stomached aliquots (surface swab specimens, fish muscle, fish organs, sliced fish) resuspended in universal preenrichment broth were incubated at 37°C for 24 h for detection of *Aeromonas* spp. (*A. caviae*, *A. hydrophila*, and *A. sobria*), *L. monocytogenes*, *Salmonella* spp., *V. cholerae*, and *V. parahaemolyticus*. A 10-µL loopful of an enriched culture in universal preenrichment broth was subcultured onto starch-ampicillin agar (Himedia, Mumbai, India), PALCAM agar (Acumedia), and xylose-lysine-desoxycholate (XLD) agar (Oxoid) for isolation of *Aeromonas* spp., *L. monocytogenes*, and *Salmonella* spp.; and onto thiosulfate-citrate-bile salt-sucrose agar (Acumedia) for isolation of and *V. cholerae* and *V. parahaemolyticus*. All selective agars were incubated at 37°C for 24 h, except for PALCAM agar, which was incubated at 37°C for 48 h.

After incubation, colonies on starch-ampicillin agar were flooded with diluted (1:20) iodine solution (Oxoid) to detect colonies with zones of clearance. Presumptive *Aeromonas* spp. colonies were streaked onto tryptic soy agar (Acumedia) for purification and biochemical confirmation by using API 20 E (bioMérieux, Marcy l'Étoile, France). Presumptive gray–green colonies surrounded by dark halos on PALCAM agar were picked for identification of *L. monocytogenes* by using a *Listeria* genus–specific PCR and a *L. monocytogenes* species–specific PCR that targeted the *prs* gene (*22*) and the *inlA* gene (*23*).

All other bacteria isolates, *Salmonella* spp. (n = 2), *V. cholerae* (n = 16), and *V. parahaemolyticus* (n = 6), were verified biochemically by using described methods (*18*). Sequence types of *Salmonella* spp. isolates were determined by using primers and Sanger sequencing, as well as comparison of sequences in the MLST database (http://mlst.warwick.ac.uk/mlst/dbs/Senterica/documents/primersEnterica_html). Sixteen *V. cholerae* isolates from 9 fish and 1 fish tank water samples were screened for virulence genes (*ctxA*, *ctxB*, and *tcpA*) by using published primers (*24–26*). Six *V. parahaemolyticus* isolates from 5 fish samples were screened for virulence genes (*tdh*, *trh1*, and *trh2*) by using published primers (*27*,*28*). Nucleic acids of all bacterial isolates, including GBS, were extracted by using the DNeasy Blood and Tissue Kit (QIAGEN). Amplified product sizes were analyzed by using

the QIAxcel DNA Screening Kit (QIAGEN) or the QIAxcel DNA High Resolution Kit (QIAGEN) as appropriate.

SPCs in Fish Pieces before and after Rinsing

We analyzed 21 sets of saltwater fish muscle samples to determine if SPC could be reduced by rinsing with water. Each set of samples consisted of 2 muscle pieces (each 5 cm × 5 cm × 1 cm). One piece served as a control (before rinsing), and the other piece was rinsed with 500 mL of sterile water. We analyzed fish muscle samples for SPCs according a described method (*18*).

References

1. van der Mee-Marquet N, Domelier A-S, Salloum M, Violette J, Arnault L, Gaillard N, et al.; Bloodstream Infection Study Group of the Reseau des Hygienistes de la Region Centre. Molecular characterization of temporally and geographically matched *Streptococcus agalactiae* strains isolated from food products and bloodstream infections. Foodborne Pathog Dis. 2009;6:1177–83. PubMed http://dx.doi.org/10.1089/fpd.2009.0287

2. Chau ML, Hartantyo SH, Yap M, Kang JSL, Aung KT, Gutiérrez RA, et al. Diarrheagenic pathogens in adults attending a hospital in Singapore. BMC Infect Dis. 2016;16:32. PubMed http://dx.doi.org/10.1186/s12879-016-1354-0

3. Lamy M-C, Dramsi S, Billoët A, Réglier-Poupet H, Tazi A, Raymond J, et al. Rapid detection of the "highly virulent" group B *Streptococcus* ST-17 clone. Microbes Infect. 2006;8:1714–22. PubMed http://dx.doi.org/10.1016/j.micinf.2006.02.008

4. Poyart C, Tazi A, Réglier-Poupet H, Billoët A, Tavares N, Raymond J, et al. Multiplex PCR assay for rapid and accurate capsular typing of group B streptococci. J Clin Microbiol. 2007;45:1985–8. PubMed http://dx.doi.org/10.1128/JCM.00159-07

5. Imperi M, Pataracchia M, Alfarone G, Baldassarri L, Orefici G, Creti R. A multiplex PCR assay for the direct identification of the capsular type (Ia to IX) of *Streptococcus agalactiae*. J Microbiol Methods. 2010;80:212–4. PubMed http://dx.doi.org/10.1016/j.mimet.2009.11.010

6. Jones N, Bohnsack JF, Takahashi S, Oliver KA, Chan M-S, Kunst F, et al. Multilocus sequence typing system for group B *Streptococcus*. J Clin Microbiol. 2003;41:2530–6. PubMed http://dx.doi.org/10.1128/JCM.41.6.2530-2536.2003

7. Kalimuddin S, Chen SL, Lim CTK, Koh TH, Tan TY, Kam M, et al.; Singapore Group B Streptococcus Consortium. 2015 epidemic of severe *Streptococcus agalactiae* ST283 infections in Singapore associated with the consumption of raw freshwater fish: a detailed analysis of clinical, epidemiological and bacterial sequencing data. Clin Infect Dis. 2017;64(Suppl_2):S145–52. PubMed http://dx.doi.org/10.1093/cid/cix021

8. Mehershahi KS, Hsu LY, Koh TH, Chen SL. Complete genome sequence of *Streptococcus agalactiae* serotype III, multilocus sequence type 283 strain SG-M1. Genome Announc. 2015;3:e01188–15. PubMed

9. Li H, Durbin R. Fast and accurate short read alignment with Burrows-Wheeler transform. Bioinformatics. 2009;25:1754–60. PubMed http://dx.doi.org/10.1093/bioinformatics/btp324

10. Wilm A, Aw PPK, Bertrand D, Yeo GHT, Ong SH, Wong CH, et al. LoFreq: a sequence-quality aware, ultra-sensitive variant caller for uncovering cell-population heterogeneity from high-throughput sequencing datasets. Nucleic Acids Res. 2012;40:11189–201. PubMed http://dx.doi.org/10.1093/nar/gks918

11. Lee T-H, Guo H, Wang X, Kim C, Paterson AH. SNPhylo: a pipeline to construct a phylogenetic tree from huge SNP data. BMC Genomics. 2014;15:162. PubMed http://dx.doi.org/10.1186/1471-2164-15-162

12. Paradis E, Claude J, Strimmer K. APE: analyses of phylogenetics and evolution in R language. Bioinformatics. 2004;20:289–90. PubMed http://dx.doi.org/10.1093/bioinformatics/btg412

13. Yu G, Smith DK, Zhu H, Guan Y, Lam TT-Y. GGTREE: an R package for visualization and annotation of phylogenetic trees with their covariates and other associated data. Methods in Ecology and Evolution. 2016 [cited 2017 Aug 16]. https://www.researchgate.net/publication/306241714_ggtree_an_R_package_for_visualization_and_annotation_of_phylogenetic_trees_with_their_covariates_and_other_associated_data

14. Inouye M, Dashnow H, Raven L-A, Schultz MB, Pope BJ, Tomita T, et al. SRST2: Rapid genomic surveillance for public health and hospital microbiology labs. Genome Med. 2014;6:90. PubMed http://dx.doi.org/10.1186/s13073-014-0090-6

15. Camacho C, Coulouris G, Avagyan V, Ma N, Papadopoulos J, Bealer K, et al. BLAST+: architecture and applications. BMC Bioinformatics. 2009;10:421. PubMed http://dx.doi.org/10.1186/1471-2105-10-421

16. Jolley KA, Maiden MC. BIGSdb: scalable analysis of bacterial genome variation at the population level. BMC Bioinformatics. 2010;11:595. PubMed http://dx.doi.org/10.1186/1471-2105-11-595

17. Gupta SK, Padmanabhan BR, Diene SM, Lopez-Rojas R, Kempf M, Landraud L, et al. ARG-ANNOT, a new bioinformatic tool to discover antibiotic resistance genes in bacterial genomes. Antimicrob Agents Chemother. 2014;58:212–20. PubMed http://dx.doi.org/10.1128/AAC.01310-13

18. Aung KT, Lo JA, Chau ML, Kang JS, Yap HM, Gutiérrez RA, et al. Microbiological safety assessment and risk mitigation of Indian rojak (deep fried ready-to-eat food) in Singapore. Southeast Asian J Trop Med Public Health. 2016;47:1231–45.

19. Rosec JP, Gigaud O. Staphylococcal enterotoxin genes of classical and new types detected by PCR in France. Int J Food Microbiol. 2002;77:61–70. PubMed http://dx.doi.org/10.1016/S0168-1605(02)00044-2

20. Cremonesi P, Luzzana M, Brasca M, Morandi S, Lodi R, Vimercati C, et al. Development of a multiplex PCR assay for the identification of *Staphylococcus aureus* enterotoxigenic strains isolated from milk and dairy products. Mol Cell Probes. 2005;19:299–305. PubMed http://dx.doi.org/10.1016/j.mcp.2005.03.002

21. Strommenger B, Kettlitz C, Werner G, Witte W. Multiplex PCR assay for simultaneous detection of nine clinically relevant antibiotic resistance genes in *Staphylococcus aureus*. J Clin Microbiol. 2003;41:4089–94. PubMed http://dx.doi.org/10.1128/JCM.41.9.4089-4094.2003

22. Doumith M, Buchrieser C, Glaser P, Jacquet C, Martin P. Differentiation of the major *Listeria monocytogenes* serovars by multiplex PCR. J Clin Microbiol. 2004;42:3819–22. PubMed http://dx.doi.org/10.1128/JCM.42.8.3819-3822.2004

23. Liu D, Lawrence ML, Wiedmann M, Gorski L, Mandrell RE, Ainsworth AJ, et al. *Listeria monocytogenes* subgroups IIIA, IIIB, and IIIC delineate genetically distinct populations with varied pathogenic potential. J Clin Microbiol. 2006;44:4229–33. PubMed http://dx.doi.org/10.1128/JCM.01032-06

24. Hoshino K, Yamasaki S, Mukhopadhyay AK, Chakraborty S, Basu A, Bhattacharya SK, et al. Development and evaluation of a multiplex PCR assay for rapid detection of toxigenic *Vibrio cholerae* O1 and O139. FEMS Immunol Med Microbiol. 1998;20:201–7. PubMed http://dx.doi.org/10.1111/j.1574-695X.1998.tb01128.x

25. Imani FA, Iman ID, Hosseini DR, Karami A, Marashi SM. Design of a multiplex PCR method for detection of toxigenic-pathogenic in *Vibrio cholerae.* Asian Pac J Trop Med. 2013;6:115–8. PubMed http://dx.doi.org/10.1016/S1995-7645(13)60005-X

26. Rivera IN, Chun J, Huq A, Sack RB, Colwell RR. Genotypes associated with virulence in environmental isolates of *Vibrio cholerae.* Appl Environ Microbiol. 2001;67:2421–9. PubMed http://dx.doi.org/10.1128/AEM.67.6.2421-2429.2001

27. Bej AK, Patterson DP, Brasher CW, Vickery MC, Jones DD, Kaysner CA. Detection of total and hemolysin-producing *Vibrio parahaemolyticus* in shellfish using multiplex PCR amplification of *tl*, *tdh* and *trh.* J Microbiol Methods. 1999;36:215–25. PubMed http://dx.doi.org/10.1016/S0167-7012(99)00037-8

28. Tada J, Ohashi T, Nishimura N, Shirasaki Y, Ozaki H, Fukushima S, et al. Detection of the thermostable direct hemolysin gene (*tdh*) and the thermostable direct hemolysin-related hemolysin gene (*trh*) of *Vibrio parahaemolyticus* by polymerase chain reaction. Mol Cell Probes. 1992;6:477–87. PubMed http://dx.doi.org/10.1016/0890-8508(92)90044-X

29. Ward RD. FISH-BOL, a case study for DNA barcodes. Methods Mol Biol. 2012;858:423–39. PubMed http://dx.doi.org/10.1007/978-1-61779-591-6_21

30. Agri-Food and Veterinary Authority of Singapore. Sale of food act, chapter 283, section 56 (1), food regulations. December 20, 2016 [cited 2017 Jan 9]. http://www.ava.gov.sg/legislation

31. Da Cunha V, Davies MR, Douarre PE, Rosinski-Chupin I, Margarit I, Spinali S, et al.; DEVANI Consortium. *Streptococcus agalactiae* clones infecting humans were selected and fixed through the extensive use of tetracycline. Nat Commun. 2014;5:4544. PubMed http://dx.doi.org/10.1038/ncomms5544

Technical Appendix Table 1. Characteristics of freshwater and saltwater fish tested during investigation of group B *Streptococcus* infections, Singapore, 2015–2016

Sampling location	Type of sample	No. samples	Type of fish collected
Noneating establishment			
Port	Whole fish	586	Freshwater fish sold as Asian bighead carp (*Hypophthalmichthys nobilis*), grass carp (*Ctenopharyngodon idella*), Pangasius dory (*Pangasius* spp.), snakehead (*Channa* spp.), and tilapia (*Oreochromis* spp.)
Fresh produce markets (fish stalls at ports and wet markets and fresh produce sections of supermarkets)	Whole fish, fish parts	39	Freshwater fish sold as Asian bighead carp, snakehead-toman (*C. micropeltes*), snakehead-haruan (*C. striata)*, silver carp (*Hypophthalmichthys molitrix*), and tilapia
Fresh produce markets (fish stalls at ports and wet markets and fresh produce sections of supermarkets)	Whole fish, fish parts	23	Saltwater fish sold as mackerel tuna (*Euthynnus affinis*), ribbonfish (*Trichiurus* spp.), salmon (*Salmo salar*), and wolf herring (*Chirocentrus* spp.)
Sashimi suppliers*	Whole fish, fish parts	21	Saltwater fish sold as salmon and tuna (*Thunnus* spp.)
Eating establishment			
Food stalls in (in hawker centers, coffee shops,and eating houses)	Sliced fish	25	Freshwater fish sold as Asian bighead carp, grass carp, Pangasius dory, and snakehead
Food stalls in (in hawker centers, coffee shops,and eating houses)	Sliced fish	21	Saltwater fish sold as salmon and Spanish mackerel (*Scomberomorus* spp.), and wolf herring.
Restaurants and snack bars*	Sliced fish	282	Saltwater fish sold as salmon, tuna, and *taï*†

*These locations did not sell freshwater fish meant for raw consumption.
†Generally refers to red sea bream (*Pagrus major*) in authentic Japanese cuisine, but it can be substituted with other fish by retailers in Singapore.

Technical Appendix Table 2. Testing of subsamples during investigation of group B *Streptococcus* infections, Singapore, 2015–2016*

	Purpose of testing			
Characteristic	Detection of organism implicated in outbreak	Detection of food safety indicators	Detection of food hygiene indicators	Measurement of total microbial load in food
Testing parameter	GBS, GBS, serotype III, and GBS ST283	*Aeromonas* spp. (*A. caviae*, *A. hydrophila*, and *A. sobria*); *Listeria monocytogenes*; *Salmonella* spp.; *Vibrio cholerae* and *V. parahaemolyticus*	*Escherichia coli* and *Staphylococcus aureus* counts	Standard plate count
Type of sample				
Whole fish, fish parts				
Organs (brain, eyes, spleen, or kidneys)	Yes	No	No	No
Surface swab specimen	Yes	Yes†	Yes†	No
Muscle	Yes	Yes†	Yes†	Yes†
Sliced fish	Yes	Yes‡	Yes	Yes
Water	Yes	Yes‡	Yes	Yes

*GBS, group B *Streptococcus*; ST, sequence type.
†These parameters were tested on whole fish/fish part samples collected at only fresh produce markets and sashimi suppliers; whole fish samples collected from ports were excluded.
‡All food safety indicators were tested except for *Aeromonas* spp.

Technical Appendix Table 3. Characteristics of group B *Streptococcus* isolates from fish and water samples, Singapore, 2015–2016*

Sample ID	No. isolates analyzed	Sample description	Type of sample	Sold as RTE dish	Sampling site†	Sold as freshwater fish	Serotype	ST	Antimicrobial drug resistance gene‡
SGEHI2015–63§	2	Fish parts (slab) sold as snakehead-toman	Muscle	No	Wet market stall	Yes	Ia	7	–
SGEHI2015-IV87	2	Sliced fish sold as salmon	Muscle	Yes (sashimi)	Restaurant/snack bar	No	Ia	7	–
SGEHI2015-IV170	2	Sliced fish sold as taï (tilapia)¶	Muscle	Yes (sashimi)	Restaurant/snack bar	No	Ia	7	–
SGEHI2015–49§	1	Fish parts (slab) sold as snakehead-toman	Surface swab	No	Wet market stall	Yes	Ia	23	*tetM*
SGEHI2015–50§	1	Fish parts (slab) sold as snakehead-toman	Muscle	No	Wet market stall	Yes	Ia	23	*tetM*
SGEHI2015–243	10	Whole fish sold asmackerel tuna	Surface swab	No	Fish stall (port)	No	Ia	23	*lsaC*, *tetM*
SGEHI2015-IV89	5	Sliced fish sold as tuna	Muscle	Yes (sashimi)	Restaurant/snack bar	No	Ia	103	*tetO#*
SGEHI2015-IV211	2	Sliced fish sold as tuna	Muscle	Yes (sashimi)	Restaurant/snack bar	No	Ia	103	–
SGEHI2015-IV232	2	Sliced fish sold as salmon	Muscle	Yes (*lo-hei*)	Restaurant/snack bar	No	Ia	103	–
SGEHI2015–77§	1	Fish part (slab) sold as snakehead-toman	Muscle	No	Wet market stall	Yes	II	28	*tetM*
SGEHI2015-II47	1	Sliced fish sold as grass carp	Muscle	Yes (*yusheng*)	Food stall	Yes	II	652	*tetL*, *tetM*
LG01	1	Whole fish sold as Asian bighead carp	Organs	No	Port	Yes	III	283	–
LG02	1	Whole fish sold as Asian bighead carp	Muscle	No	Port	Yes	III	283	–
LG03	1	Whole fish sold as Asian bighead carp	Surface swab	No	Port	Yes	III	283	–
LG04	1	Whole fish sold as Asian bighead carp	Surface swab	No	Port	Yes	III	283	–
LG05	1	Whole fish sold as Asian bighead carp	Surface swab	No	Port	Yes	III	283	–
LG06	1	Whole fish sold as Asian bighead carp	Organs	No	Port	Yes	III	283	–
LG07	1	Water for holding live freshwater fish	NA	NA	Port	NA	III	283	–
LG08	1	Water for holding live freshwater fish	NA	NA	Port	NA	III	283	–
LG09	1	Water for holding live freshwater fish	NA	NA	Port	NA	III	283	–
LG10	1	Water for holding live freshwater fish	NA	NA	Port	NA	III	283	–
LG11	1	Water for holding live freshwater fish	NA	NA	Port	NA	III	283	–

Sample ID	No. isolates analyzed	Sample description	Type of sample	Sold as RTE dish	Sampling site†	Sold as freshwater fish	Serotype	ST	Antimicrobial drug resistance gene‡
LG12	1	Water for holding live freshwater fish	NA	NA	Port	NA			–
SGEHI2015-NWC941§	3	Sliced fish sold as grass carp	Muscle	Yes (*yusheng*)	Food stall	Yes	III	283	–
SGEHI2015–107§	1	Whole live fish sold as snakehead-haruan	Muscle	No	Wet market stall	Yes	III	283	–
SGEHI2015–95§	1	Fish part (slab) sold as silver carp	Muscle	No	Supermarket	Yes	III	283	–
SGEHI2015–22§	2	Fish part (tail) sold as Asian bighead carp	Muscle	No	Fish stall (port)	Yes	III	283	–
SGEHI2015–25§	1	Fish part (head) sold as Asian bighead carp	Muscle	No	Fish stall (port)	Yes	III	283	–
SGEHI2015–51§	1	Fish part (head) sold as Asian bighead carp	Surface swab	No	Supermarket	Yes	III	283	–
SGEHI2015–31§	4	Fish part (slab) sold as snakehead-toman	Muscle	No	Fish stall (port)	Yes	III	283	–
SGEHI2015–57§	1	Whole fish sold as black tilapia	Muscle	No	Wet market stall	Yes	III	283	–
SGEHI2015–101§	1	Whole fish sold as black tilapia	Muscle	No	Wet market stall	Yes	III	283	–
SGEHI2015–113§	1	Whole fish sold as black tilapia	Muscle	No	Supermarket	Yes	III	283	*tetM*
SGEHI2015–114§	1	Whole fish sold as black tilapia	Organs	No	Supermarket	Yes	III	283	*tetM*
SGEHI2015–29§	2	Whole fish sold as red tilapia	Organs	No	Fish stall (port)	Yes	III	283	–
SGEHI2015–59§	1	Whole fish sold as black tilapia	Surface swab	No	Wet market stall	Yes	III	283	–
SGEHI2015–60§	1	Whole fish sold as black tilapia	Muscle	No	Wet market stall	Yes	Ia	24	*tetM*
SGEHI2015-II33	1	Sliced fish sold as snakehead	Muscle	Yes (*yusheng*)	Food stall	Yes	III	335	*tetM*
SGEHI2015-IV45	5	Sliced fish sold as taï (tilapia)¶	Muscle	Yes (sashimi)	Restaurant/snack bar	No	III	651	*ermB*, *tetL*
SGEHI2015-IV118	2	Sliced fish sold as salmon	Muscle	Yes (sashimi)	Restaurant/snack bar	No	III	861	*lsaC*, *msrD*, *tetO*
SGEHI2015-IV100	3	Sliced fish sold as salmon	Muscle	Yes (sashimi)	Restaurant/snack bar	No	V, Ia	1, 485	*tetM*
SGEHI2015-IV104	2	Sliced fish sold as salmon	Muscle	Yes (sashimi)	Restaurant/snack bar	No	V	1	*tetM*
SGEHI2015-II55	2	Sliced fish sold as wolf herring	Muscle	Yes (*yusheng*)	Food stall	No	V	1	*tetM*
SGEHI2015-II56	1	Sliced fish sold as Asian bighead carp	Muscle	No	Food stall	Yes	V	1	*tetM*
SGEHI2015-IV74	1	Sliced fish sold as salmon	Muscle	Yes (sashimi)	Restaurant/snack bar	No	VII	1	*tetM*

Sample ID	No. isolates analyzed	Sample description	Type of sample	Sold as RTE dish	Sampling site†	Sold as freshwater fish	Serotype	ST	Antimicrobial drug resistance gene‡
SGEHI2015-IV72	2	Sliced fish sold as salmon	Muscle	Yes (sashimi)	Restaurant/snack bar	No	VII	1	*tetM*, *tetO*
SGEHI2015-IV132	2	Sliced fish sold as salmon	Muscle	Yes (sashimi)	Restaurant/snack bar	No	V	24	*tetM*
SGEHI2015-IV227	1	Sliced fish sold as wolf herring	Muscle	Yes (*lo-hei*)	Restaurant/snack bar	No	VI	167	*tetM*

*NA, not applicable; RTE, ready-to-eat; ST, sequence type; *lo-hei*, Lunar New Year festive dish consisting of raw fish slices served with raw vegetables and condiments; *yusheng*, Chinese style sliced raw fish dish served separately with porridge.
†Food stalls refer to stalls housed within larger eating establishments that include hawker centers, coffee shops, and eating houses. Supermarkets refer to fresh produce sections of supermarkets and exclude sashimi and sushi counters of supermarkets.
‡–, no genes encoding resistance from the ARGannot database (including those acting on aminoglycosides, β-lactams, colistin, fosfomycin, fluoroquinolones, glycopeptides, macrolides, lincosamide, streptogramines, phenicols, rifampin, sulfonamides, tetracyclines, and trimethoprim) were detected by SRST2 from the genome sequencing data.
§Isolates also described in a study (*7*) on the analysis of clinical, epidemiologic, and bacterial sequencing data obtained during investigation of group B *Streptococcus* infections.
¶Fish identified as tilapia (*Oreochromis* spp.) by using the DNA barcoding method (*29*).
#Gene encoding resistance to tetracycline (t*etO*) was detected in 3 of the 5 isolates.

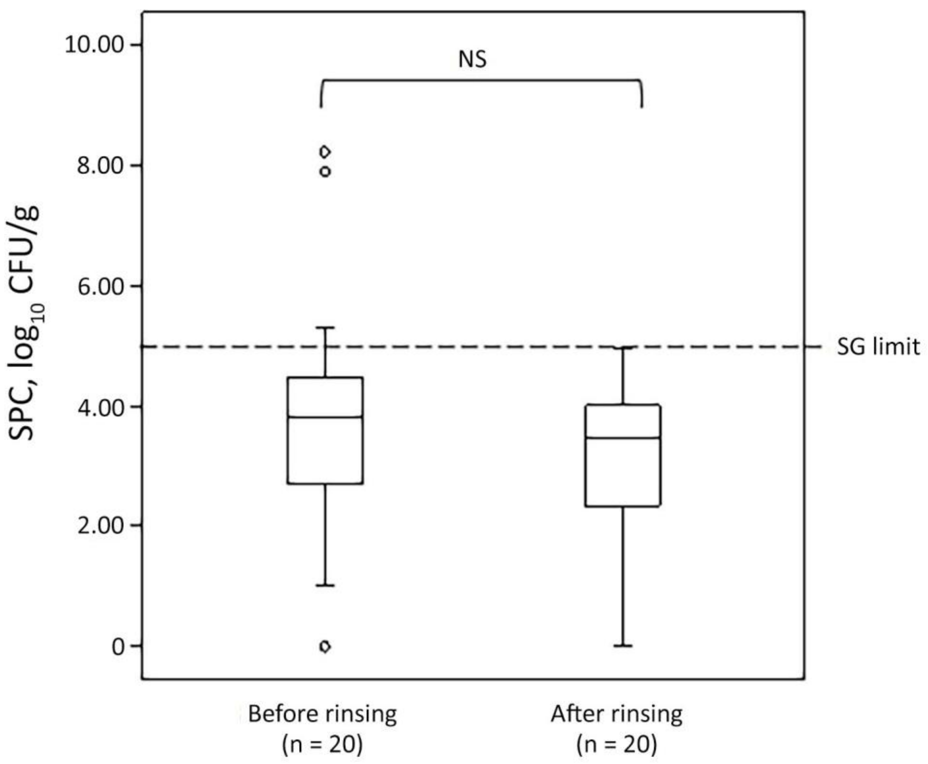

Technical Appendix Figure 1. Group B *Streptococcus* infections caused by handling and consumption of raw fish, Singapore, 2015–2016. SPC of fish muscle before and after rinsing with water. Dashed horizontal line indicates regulatory limit of Singapore for SPCs in ready-to-eat foods (<5 log_{10} CFU/g) (*30*). Top and bottom of boxes in plots indicate 25th and 75th percentiles, horizontal lines indicate medians, and error bars indicate minimum and maximum values. Open circles indicate outliers. NS, not significant (p>0.05); SPC, standard plate count; SG, Singapore government.

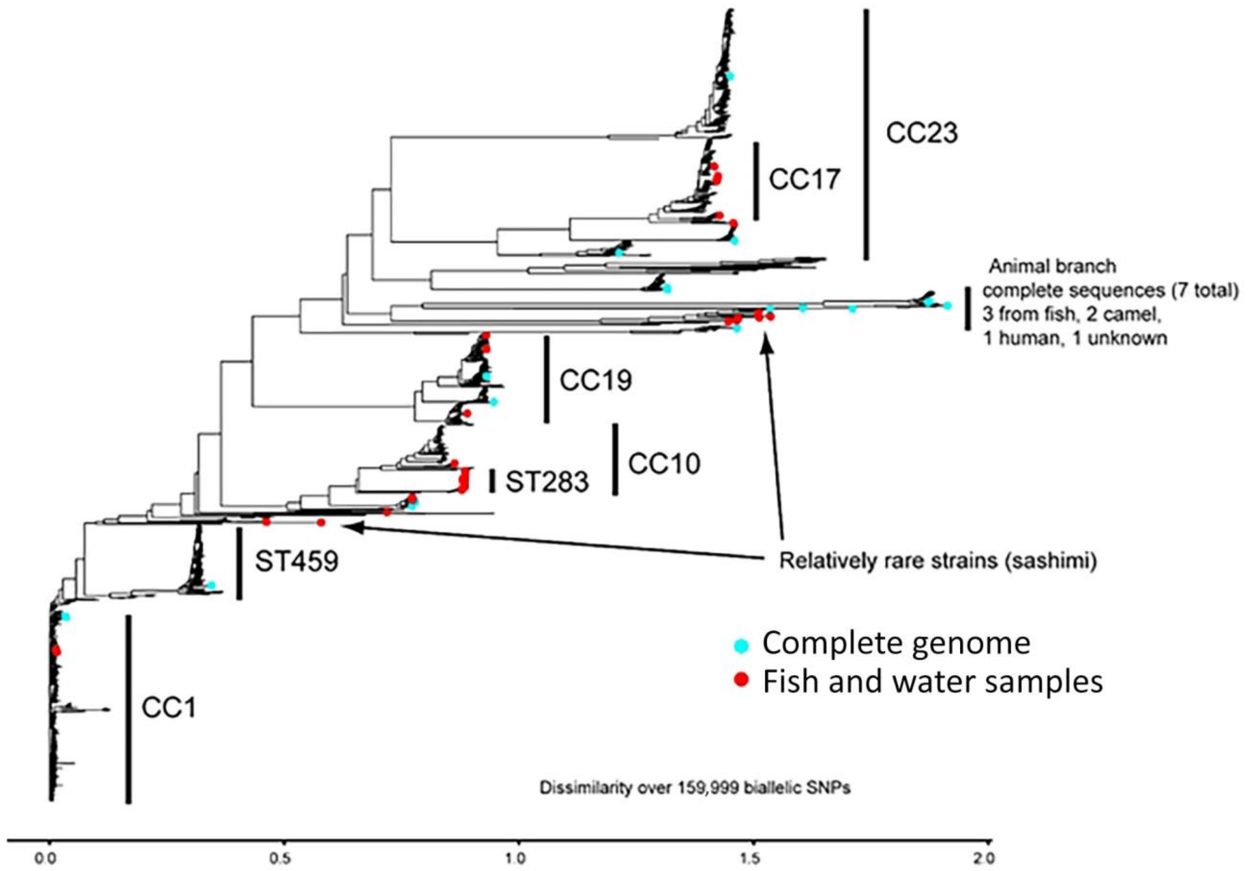

Technical Appendix Figure 2. Group B *Streptococcus* infections caused by handling and consumption of raw fish, Singapore, 2015–2016. Global phylogenetic tree (neighbor-joining tree) with SNPs relative to the SG-M1 reference genome for 1,369 GBS strains. Red circles indicate strains isolated from fish and fish tank water samples sequenced during this study. Blue circles indicate complete publicly available genome sequences. All other strains are sequences from the GenBank Short Read Archive. Vertical black bars indicate CCs (*31*) and STs. Black arrows indicate several relatively rare fish strains from this study that fall outside the major CCs and have relatively few neighboring publicly available sequences. Seventeen of these strains (just below CC17) are placed among a set of strains that are more likely not to have come from humans (fish and camel strains). CC, clonal complex; GBS, group B *Streptococcus*; SNPs, single-nucleotide polymorphisms; ST, sequence type.
